# A comparison of advanced semi-quantitative amyloid PET analysis methods

**DOI:** 10.1007/s00259-022-05846-1

**Published:** 2022-06-02

**Authors:** Enrico Peira, Davide Poggiali, Matteo Pardini, Henryk Barthel, Osama Sabri, Silvia Morbelli, Annachiara Cagnin, Andrea Chincarini, Diego Cecchin

**Affiliations:** 1 INFN – National Institute of Nuclear Physics, via Dodecaneso 33, 16146 Genoa, Italy; 2 Department of Neuroscience, Rehabilitation, Ophthalmology, Genetics, Child and Maternal Health (DINOGMI), University of Genoa, Genoa, Italy; 3 PNC - Padua Neuroscience Center, University of Padua, Padua, Italy; 4 IRCCS Ospedale Policlinico San Martino, Genoa, Italy; 5 Department of Nuclear Medicine, University Hospital Leipzig, Leipzig, Germany; 6 Nuclear Medicine Unit, Department of Health Sciences (DISSAL), University of Genoa, Genoa, Italy; 7 Neurology Unit, Department of Neurology, University Hospital of Padua, Padua, Italy; 8 Nuclear Medicine Unit, Department of Medicine - DIMED, University Hospital of Padua, Padua, Italy

**Keywords:** Amyloid PET, Quantification, Regional amyloid, Dual time point, Age-related amyloid

## Abstract

**Purpose:**

To date, there is no consensus on how to semi-quantitatively assess brain amyloid PET. Some approaches use late acquisition alone (e.g., ELBA, based on radiomic features), others integrate the early scan (e.g., TDr, which targets the area of maximum perfusion) and structural imaging (e.g., WMR, that compares kinetic behaviour of white and grey matter, or SI based on the kinetic characteristics of the grey matter alone). In this study SUVr, ELBA, TDr, WMR, and SI were compared. The latter — the most complete one — provided the reference measure for amyloid burden allowing to assess the efficacy and feasibility in clinical setting of the other approaches.

**Methods:**

We used data from 85 patients (aged 44–87) who underwent dual time-point PET/MRI acquisitions. The correlations with SI were computed and the methods compared with the visual assessment. Assuming SUVr, ELBA, TDr, and WMR to be independent measures, we linearly combined them to obtain more robust indices. Finally, we investigated possible associations between each quantifier and age in amyloid-negative patients.

**Results:**

Each quantifier exhibited excellent agreement with visual assessment and strong correlation with SI (average AUC = 0.99, *ρ* = 0.91). Exceptions to this were observed for subcortical regions with ELBA and WMR (*ρ*_ELBA_ = 0.44, *ρ*_WMR_ = 0.70). The linear combinations showed better performances than the individual methods. Significant associations were observed between TDr, WMR, SI, and age in amyloid-negative patients (*p* < 0.05).

**Conclusion:**

Among the other methods, TDr came closest to the reference with less implementation complexity. Moreover, this study suggests that combining independent approaches gives better results than the individual procedure, so efforts should focus on multi-classifier systems for amyloid PET. Finally, the ability of techniques integrating blood perfusion to depict age-related variations in amyloid load in amyloid-negative subjects demonstrates the goodness of the estimate.

**Supplementary Information:**

The online version contains supplementary material available at 10.1007/s00259-022-05846-1.

## Introduction

Amyloid PET (amy-PET) is an imaging technique that enables highly accurate, in vivo detection of amyloid-β (Aβ) plaques, considered a pathological hallmark of Alzheimer’s disease (AD) [1]. Over the years, amy-PET has provided useful support to clinicians by increasing diagnostic confidence and helping them refine management plans [2, 3]. In clinical practice, amy-PET is mainly inspected qualitatively, sometimes with the aid of structural imaging (i.e., CT or MRI), with results classified as positive or negative. In terms of diagnosing AD, the negative predictive value of the test is very high, whereas the implications of a positive result are more complex and depend also on the prevalence of brain amyloidosis in the elderly. Several studies have reported Aβ deposition to be common in cognitively healthy subjects in late adulthood, and to increase in its frequency with age [4–7].

Semi-quantitative or quantitative approaches are increasingly being used to complement qualitative assessments. These measures are essential to much of the research on neurodegeneration as they improve agreement between raters [8–10], are part of the inclusion criteria (and outcome biomarkers) in anti-amyloid clinical trials [11–15], and provide valuable information on Aβ distribution that may be useful in staging the progress of an individual’s Aβ pathology [16–18].

To date, there is no established consensus on how to semi-quantitatively assess amy-PET. Besides SUVr, the most widely used [19] method, various alternatives using different sources of information are available. Analysis can be based on standard late acquisition alone, as with methods like ELBA or Aβ_L_ [20, 21], or can include tracer kinetic information obtained by adding early acquisition, as with TDr [22]. Our group proposed a more sophisticated procedure, called the Slope Index (SI), which also takes into consideration atrophy and spillover by including MRI data [23].

In this study, we compare various semi-quantitative approaches with increasing degrees of refinement at both the global and regional levels: SUVr, ELBA, TDr, WMR, and SI. In the absence of absolute quantification, we selected SI — the most complete approach — as the reference measure of Aβ load. The choice of a reliable measure as reference allowed us to assess the efficacy of the quantifiers and the feasibility of using them in clinical and research settings. Although kinetic modeling is optimal for accurate therapy monitoring and longitudinal studies [24] (in this case, a valuable compromise is made between accuracy and simplicity [25]), advanced semi-quantitative approaches (especially using dual time-window protocols) might be an option if a certain error is acceptable. The results of the present study are potentially of great importance in view of anti-amyloid treatments in patients with AD. While it can be argued that amyloid plaque load, as measured by amy-PET with standard late acquisition, is a valid surrogate endpoint for drug effects, it should also be noted that advanced semi-quantitative methods (such as those discussed in this paper) that also include blood flow analysis (using early phase) are able to detect not only amyloid load [26] but also neurodegeneration (as ^18^F-FDG does), and therefore represent a more robust end point for monitoring disease-modifying drugs targeting amyloid deposition.

## Materials and methods

### Dataset

In this study, we used a single-center dataset to test different semi-quantitative approaches, including dual time-window protocols. The data were obtained from 85 patients (aged 44–87, *μ* = 70.9 ± 10.1; 45.8% women) at the University Hospital of Leipzig, Germany. The subjects are described in detail in [23], and their clinical diagnoses are listed in Supplementary Table 1.

### PET/MRI acquisition

Each patient received an intravenous injection of ~300 MBq ^18^F-florbetaben in an integrated 3T PET/MRI system (Biograph mMR; Siemens), then underwent PET/MRI with scans performed at 0 to 10 min (early) and at 90 to 110 min (late) after injection. Late acquisition was in accordance with the recommendations of the tracer manufacturer [27] and the guidelines of the European Association of Nuclear Medicine and the Society of Nuclear Medicine [28]. Anatomical data were also obtained via 3DT1 1-mm isotropic MRI in parallel with the PET scan. Further details on the 3DT1 MRI acquisition, and the amy-PET reconstructions and correction are provided elsewhere [23]. The 85 late scans were visually inspected by two independent nuclear medicine experts and classified as either amyloid-negative (54 subjects, aged 44–87, *μ* = 69.9 ± 10.6) or amyloid-positive (31 subjects, aged 48–83, *μ* = 72.3 ± 9.1). Discrepancies were resolved by consensus discussion with a third independent reviewer.

### Image processing

Each amy-PET was semi-quantitatively assessed by means of five independent approaches (hereinafter referred to as *quantifiers*): SUVr [19], ELBA [20], TDr [22], WMR [23], and SI [23]. Details of each quantifier can be found in their respective papers. A summary of their underlying methodologies is given here:

SUVr is calculated as the ratio of count densities between a target and a reference region of interest (ROI) [19]. In this work, it was normalized (as is frequently the case in the literature) using the whole cerebellum [24].

ELBA is a radiomic-based, SUVr-independent approach designed to capture intensity distribution patterns, which are global properties of the whole brain and do not require a reference ROI [20].

TDr is defined in [22] and is the ratio of counts exploiting the information on tracer kinetics provided by dual time-point acquisition to adapt both the target and the reference ROIs of each individual.

The SI and WMR indices are obtained from an analytical method that requires dual time-point amy-PET acquisition and a co-registered MR, allowing for blood flow and partial volume effect corrections (PVEC) [23].

The SI uses a kinetic assessment of the gray matter (GM) characteristics that considers a surrogate for blood flow (through the early acquisition) including a partial correction for blood flow dependence in addition to corrections for atrophy and spillover.

The WMR instead has no immediate pathophysiological justification. It stems from the empirical observation that the intensity contrast in the early acquisition vs. the late one — calculated on both cortical and white matter (WM) regions — has distinct behavior in clearly positive and negative subjects. WMR does not involve normalization on a reference region, but the ratio is calculated on the difference between the mean activity concentration in the target region and the WM, using both the early and late scans.

Each quantifier is designed to capture specific characteristics of the image data that are directly or indirectly related to the expected amyloid load (and blood flow). These methods make use of different types of information, details of which are shown in Table 1 along with the minimum requirements to perform the analysis.

**Table 1:** Minimum requirements for each quantifier

	Acquisition			Processing	
	PET late	PET early	MRI T13D	reference ROI	target ROI
SUVr	•			•	•
ELBA	•				
TDr	•	•			
WMR	•	•	•	•	•
SI	•	•	•	•	•

Subcortical volumes, segmentation of the subcortical WM, and cortical thickness and surface area were estimated from the 3DT1 MRI using FreeSurfer 5.3 (https://surfer.nmr.mgh.harvard.edu) [29]. This processing included motion correction, skull stripping, registration to Talairach space, segmentation, intensity normalization, and parcellation mapping according to the Desikan-Killiany cortical labelling protocol.

In this study, we compared the quantifiers at both the global and regional levels. Five lobar ROIs for each hemisphere were obtained from the FreeSurfer parcellation (i.e., the frontal, parietal, temporal, occipital, and central structures); see Supplementary Fig. 1. The global ROI was obtained by merging the 10 lobar ROIs. The global and lobar ROIs were used as target regions in the analyses.

For the SI and WMR quantifiers, image registration to the MNI space was guided by the individual patient’s 3DT1, which resulted in the atlas ROIs accurately overlapping with those of the patients. For SUVr, ELBA, and TDr — since an MRI is not required — image registration was guided only by a generic amyloid template in the MNI space (see [20]), resulting in a coarser alignment between the atlas and the patients’ PET dataset.

The results from each quantifier were *z*-scored to enable better comparison of the different methods with possibly different scales.

### Image analysis

Due to the lack of an absolute quantification (full kinetic acquisition was not available in our dataset), the SI quantifier — the most comprehensive and sophisticated approach — was used as the reference measure for Aβ burden. SI includes correction for atrophy, spillover, and blood flow dependence, and is therefore the quantifier that takes the most error sources into consideration.

To compare both the global and lobar SUVr, ELBA, TDr, and WMR with SI, we first determined the correlations and quantified the dispersion with a Bland–Altman analysis.

The discriminating power of the different approaches compared with the visual assessment was then measured by the area under the receiver operating characteristic curve (AUC-ROC). Assuming SUVr, ELBA, TDr, and WMR to be proxy measures of the true Aβ load (estimated from the SI), we linearly combined them to obtain more robust indices, and compared these combinations with SI.

Finally, we assessed the sensitivity of each quantifier to Aβ plaque accumulation in patients classified qualitatively as amyloid-negative, with the idea that a more sensitive method could better identify an Aβ load that was physiologically increasing with age.

#### Assessment of the differences between the quantifiers and SI

As noted, the agreement between SUVr, ELBA, TDr, and WMR and SI was assessed with a Bland–Altman analysis. The divergences were quantified by the σ of the difference between the global and the lobar SI and the corresponding values of the other quantifiers. The confidence intervals for the σ were obtained from a 1000 iteration bootstrap procedure.

We linearly combined SUVr, ELBA, TDr, and WMR into three scores: AVG1 (the weighted mean of SUVr and ELBA), AVG2 (the weighted mean of SUVr, ELBA and TDr), and AVG3 (the weighted mean of SUVr, ELBA, TDr, and WMR). The inverse of the global divergences from SI (1/σ) were used as weights for the average mean of the quantifiers of concern. Thus, the quantifier having a better agreement with SI contributed more to each combination.

The linear correlation between the global and regional SUVr, ELBA, TDr, WMR, AVG1, AVG2, and AVG3 and the corresponding SI was measured by the Pearson correlation coefficient.

To verify SUVr, ELBA, TDr, and WMR as independent measures of Aβ load, we looked at the residuals of all possible linear models including these measures (i.e., SUVr ~ ELBA, SUVr ~ TDr, SUVr ~ WMR, ELBA ~ TDr, ELBA ~ WMR, and TDr ~ WMR). The Pearson correlation coefficient between the residuals and the predictors was estimated for each model.

#### Agreement between the quantifiers and the visual classification

The discriminating power of the quantifiers and of their combinations were measured by AUC for negative- vs. positive-labeled scans. This assessment was repeated for both the global and lobar scores. The generalized performance of each score was estimated using a 1,000 iteration bootstrap procedure.

#### Comparisons in amyloid-negative patients

Linear regression was used to test for possible associations between each quantifier (global and lobar scores), age, and cortical thickness in amyloid-negative patients.

Before running the regressions, the variance inflation factor (VIF) was computed to verify the possible collinearity between age and cortical thickness (global and lobar).

## Results

### Analysis of the differences between the quantifiers and SI

**Fig. 1:**
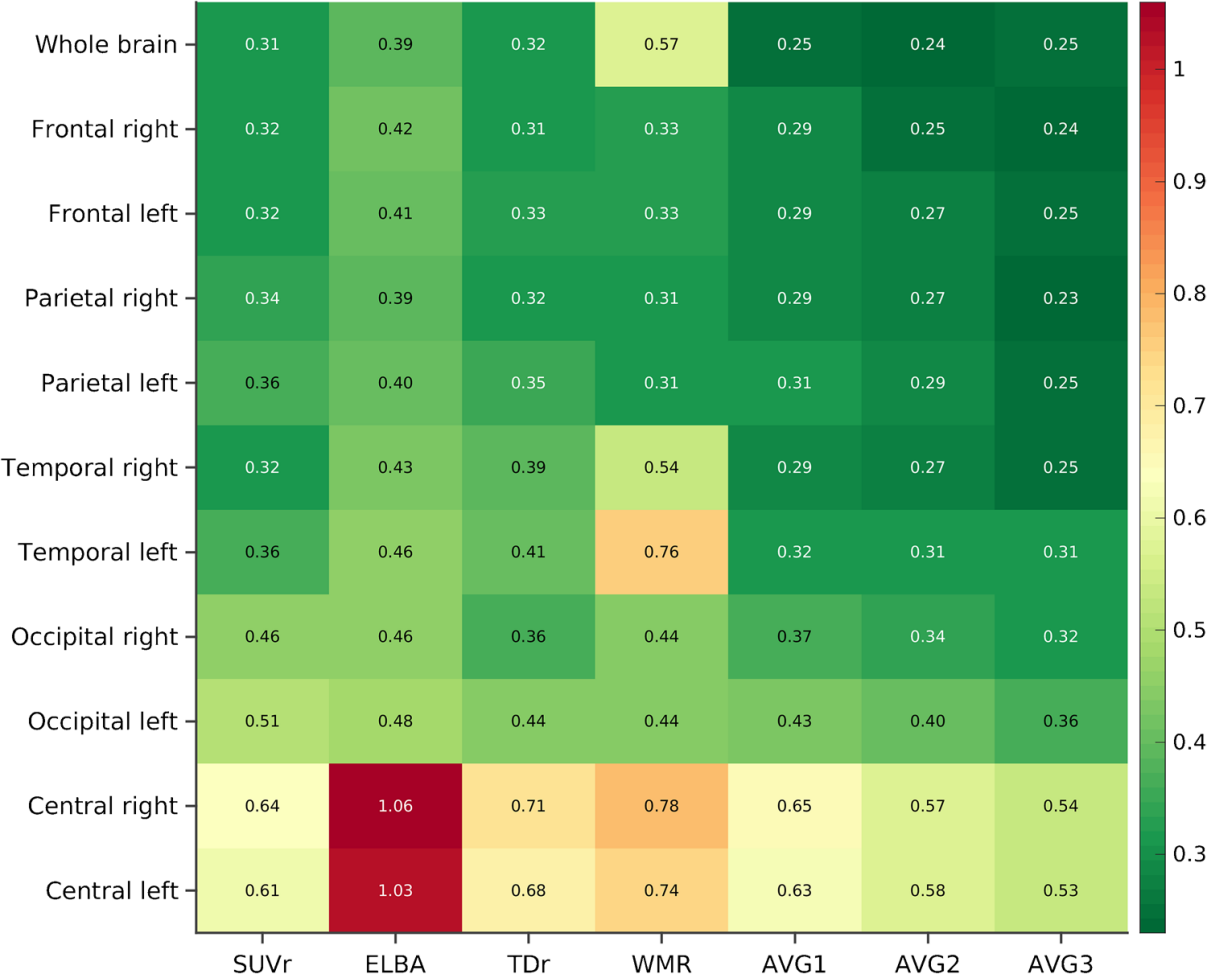
Dispersions of the quantifiers (and of their linear combinations) from SI at the brain and lobar levels. The values reported correspond to the bootstrapped divergences from SI and are expressed as the average *σ* from the Bland–Altman analysis

**Fig. 2:**
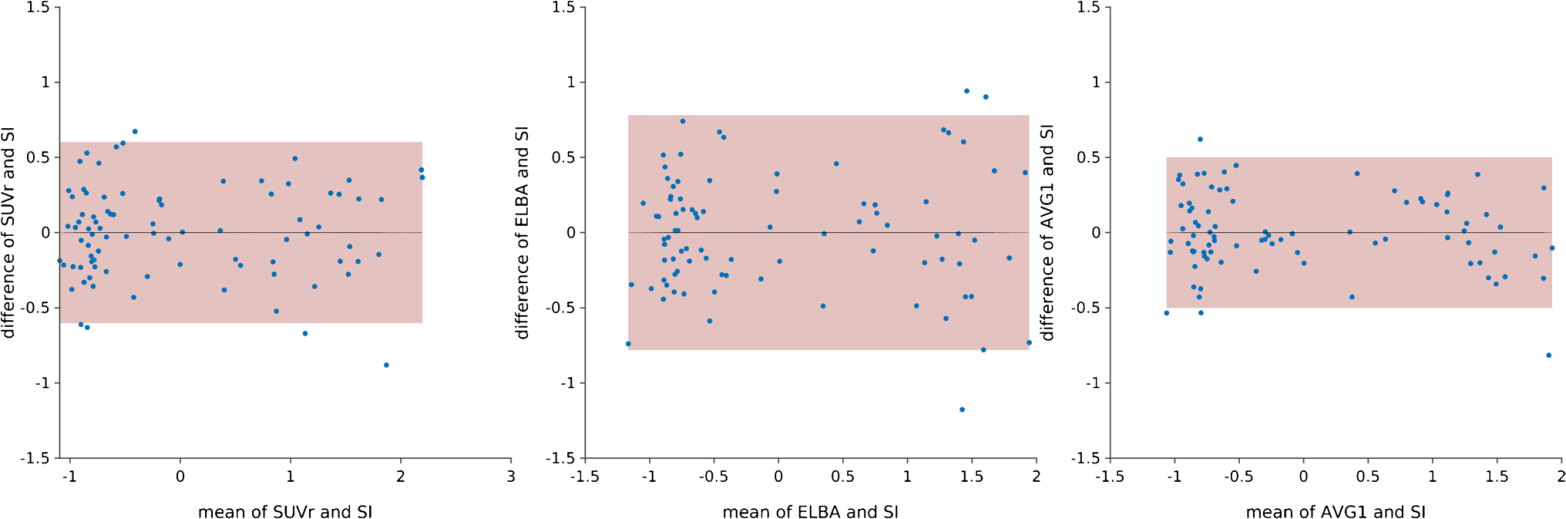
Bland–Altman plots of SUVr vs. SI (left), ELBA vs. SI (middle), and AVG1 (weighted mean of SUVr and ELBA) vs. SI (right). The quantifiers are compared in these plots at the whole-brain level. As expected, a combination of two methods (AVG1) reduces the dispersion (red area) of the Bland–Altman plot compared with the single methods

The average divergences of each quantifier from SI are summarized in Fig. 1 (the corresponding 95% confidence intervals are reported in Supplementary Table 2). In each Bland–Altman plot examined, the regression lines and the zero bias line fell within the 95% confidence interval, thus excluding bias changes over the measuring interval. Examples of the Bland–Altman plots and the divergences from SI are given in Fig. 2. Among the quantifiers, SUVr and TDr exhibited lower dispersion from SI both globally (whole brain; σ_SUVr_ = 0.31, σ_TDr_ = 0.32) and regionally (average over lobes; σ_SUVr_ = 0.42, σ_TDr_ = 0.43). The highest dispersion from SI at the global level was exhibited by WMR (whole brain *σ* = 0.57), and at the regional level by ELBA (average lobar *σ* = 0.55). Examples of cases with greater distance to the SI are shown in the Supplementary Fig. 2. The lowest lobar variances (*σ* = 0.31) were observed in TDr (frontal right lobe) and WMR (parietal right lobe). This is in line with the results that showed the frontal and parietal to be the lobes with the lowest dispersion (averages over quantifiers; *σ*_frontal_ = 0.35, *σ*_parietal_ = 0.35). On the other hand, ELBA in the right subcortical ROI exhibited the highest dispersion (*σ* = 1.06); the highest variances with SI were also observed in this region (average over quantifiers; *σ*_central_ = 0.78).

As expected, the *σ* of the three linear combinations (i.e., AVG1, AVG2, and AVG3) was lower than those of the quantifiers included in each combination (whole brain; *σ*_AVG1_ = 0.25, *σ*_AVG2_ = 0.24, *σ*_AVG3_ = 0.25; see Fig. 2). Moreover, the regional average *σ* of the linear combinations decreased as another quantifier was added.

**Fig. 3:**
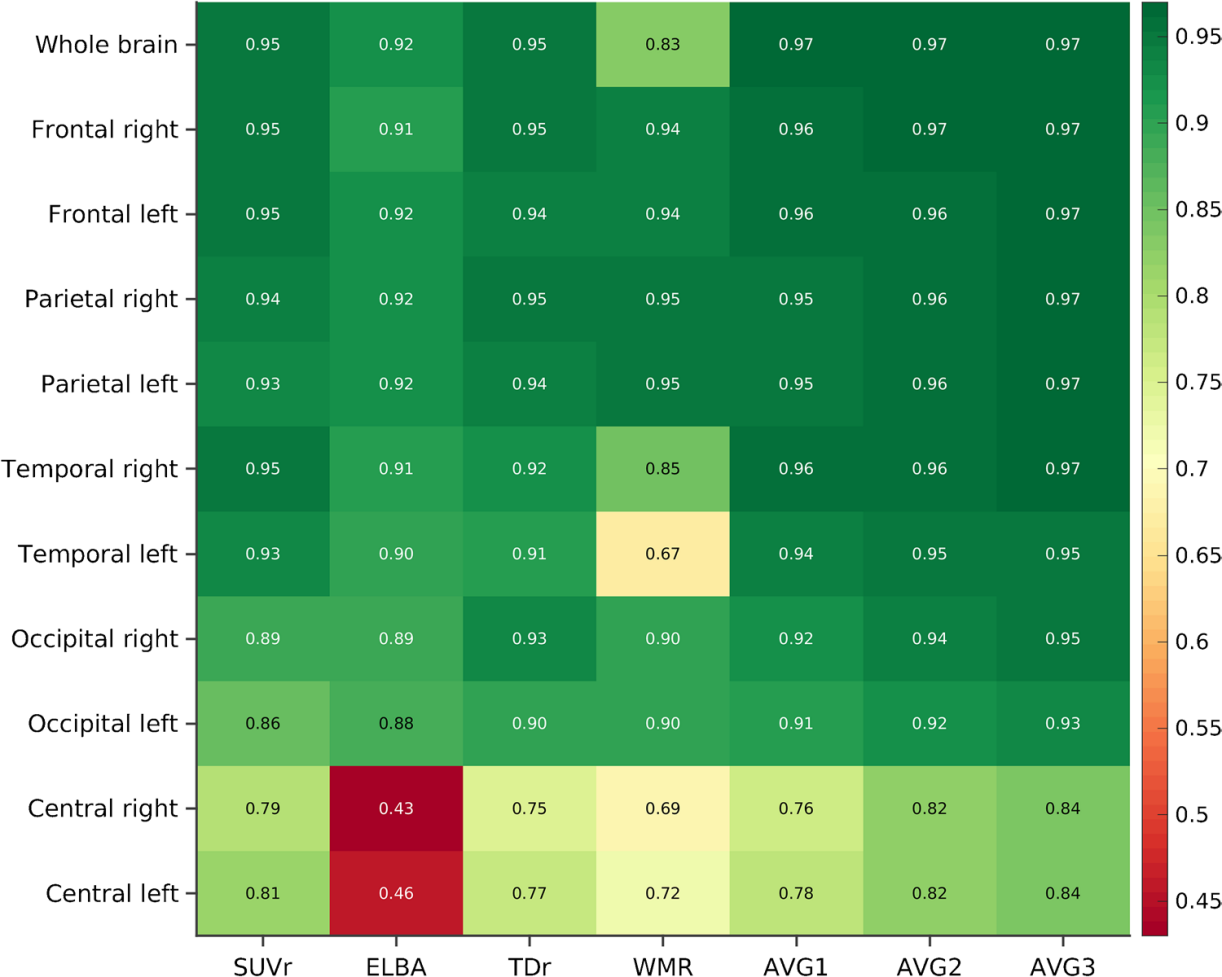
Correlations between the quantifiers (and their linear combinations) and SI at the whole-brain and lobar levels (all correlations significant at *p* < 0.05)

As evidenced by the correlation coefficients (*ρ*) summarized in Fig. 3, each quantifier correlated strongly with SI both globally and regionally, although with some exceptions. In line with the dispersion analysis, there was only a moderate correlation in the subcortical regions (average *ρ*_central_ = 0.67). Moderate correlations were also observed between SI and WMR (right subcortical *ρ* = 0.69; left temporal *ρ* = 0.67), and between SI and ELBA (central ROI; *ρ*_right_ = 0.43, *ρ*_left_ = 0.46). The strongest correlations were found in the frontal and parietal lobes (average over quantifiers; *ρ*_frontal_ = 0.94, *ρ*_parietal_ = 0.94). Consistent with the dispersion analysis, SUVr and TDr exhibited the strongest correlations with SI both globally (whole brain; *ρ*_SUVr_ = 0.95, *ρ*_TDr_ = 0.95) and regionally (average over lobes; *ρ*_SUVr_ = 0.9, *ρ*_TDr_ = 0.9). Nonetheless, the lowest correlations were with WMR at the whole-brain level (*ρ* = 0.83), and with ELBA at the regional level (average over lobes; *ρ* = 0.81). Also consistent with the dispersions, the coefficients *ρ* of the three linear combinations were higher than those of the single quantifiers included in each combination (at both the regional and global levels).

The residuals and the predictor variables of the linear models — SUVr ~ ELBA, SUVr ~ TDr, SUVr ~ WMR, ELBA ~ TDr, ELBA ~ WMR, and TDr ~ WMR — were found to be uncorrelated, while the linear regressions of the residuals of each model against the respective predictor were all found to be compatible with the null model.

### Agreement with the visual classification

**Fig. 4:**
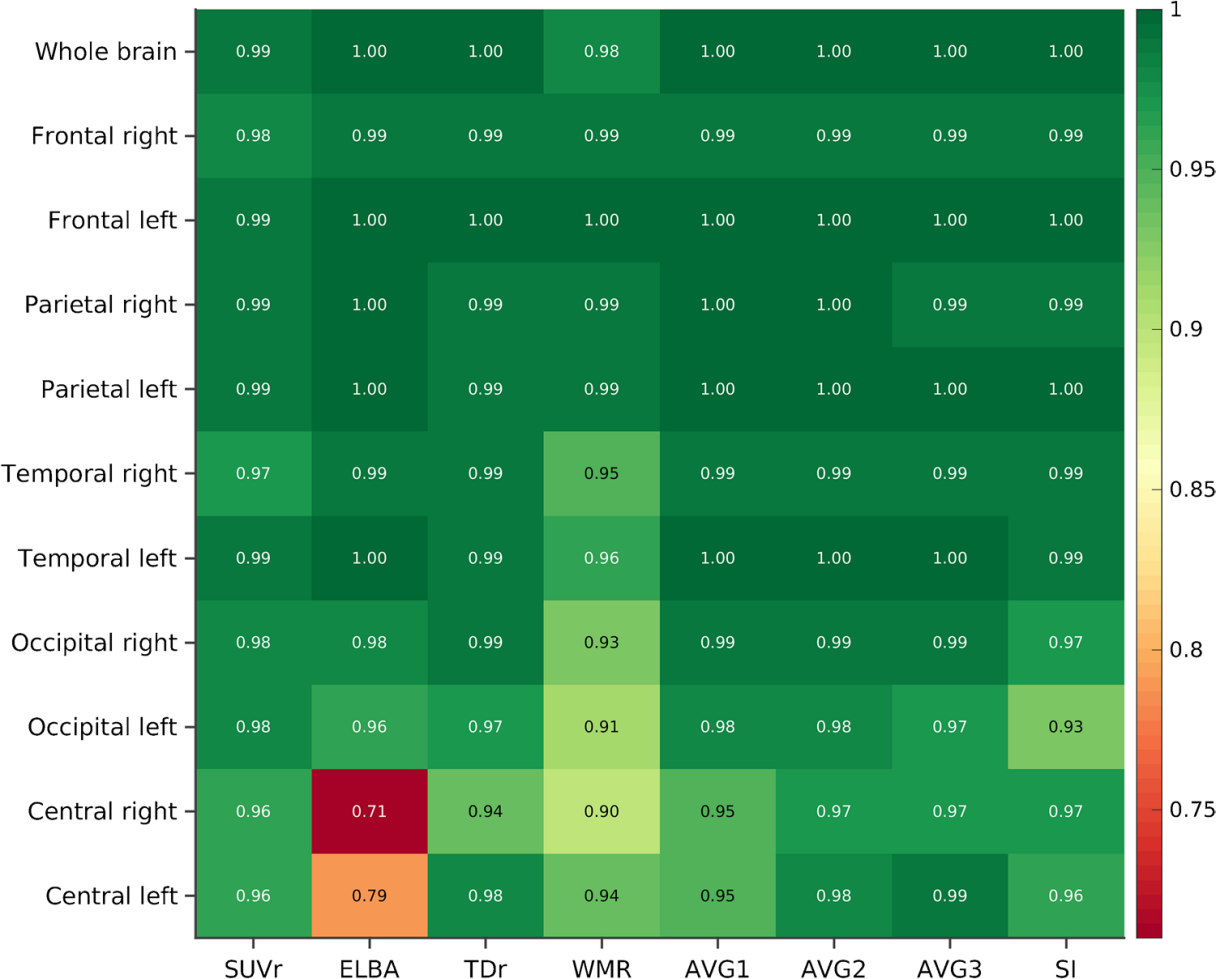
Regional and whole-brain AUC performance (average over bootstrap sampling) of the quantifiers and their linear combinations vs. visual assessment

The bootstrapped generalized performance vs. the consensus binary visual assessment is summarized in Fig. 4 and in Supplementary Table 3. The results were excellent for all the approaches (whole-brain average AUC = 0.99), and for their weighted averages (whole-brain average AUC = 1).

The lowest AUCs were observed in the subcortical lobes with ELBA (AUC_left_ = 0.79, AUC_right_ = 0.71), whereas regional AUC = 1 was observed for different quantifiers in different regions (e.g., parietal, frontal, temporal). The central was the brain area with the lowest AUC (average AUC over the quantifiers = 0.90), whereas the parietal and frontal had the highest (average AUC over the quantifiers = 0.99). With a global value of 0.98, WMR had a slightly lower AUC than the other quantifiers. However, in interpreting these results it should be borne in mind that the slight differences in the average AUCs are not relevant if the confidence intervals overlap.

### Comparisons in amyloid-negative patients

A moderate correlation between age and cortical thickness was observed in amyloid-negative patients (|*ρ*|< 0.48, *p* < 0.05). However, the VIF excluded collinearity of these variables (< 1.29 for all covariates).

The results from the linear models are given in Table 2. Below, we describe the significant associations that survived a Benjamini–Hochberg correction for multiple comparisons.

**Table 2:** Associations between the quantifier scores, age, and cortical thickness at the whole-brain and lobar levels in qualitatively assessed amyloid-negative patients

Region	Quantifier	Age		Thickness	
		*β*	*p*	*β*	*p*
Whole brain	SUVr		n.s		n.s
	ELBA		n.s		n.s
	TDr	0.021	**^a^		n.s
	WMR	0.022	*^a^		n.s
	SI	0.020	**^a^	0.724	*
Frontal right/left	SUVr		n.s./n.s		n.s./n.s
	ELBA		n.s./n.s		n.s./n.s
	TDr	0.019/0.020	**^a^/**^a^		n.s./n.s
	WMR	0.017/0.016	*^a^/*^a^		n.s./n.s
	SI	0.018/0.019	**^a^/**^a^	–/0.698	n.s./*
Parietal right/left	SUVr		n.s./n.s		n.s./n.s
	ELBA		n.s./n.s		n.s./n.s
	TDr	0.018/0.020	*^a^/**^a^		n.s./n.s
	WMR	0.014/0.013	*^a^/*^a^		n.s./n.s
	SI	0.019/0.019	**^a^/** ^a^		n.s./n.s
Temporal right/left	SUVr	0.017/–	*^a^/n.s	0.859/–	*/n.s
	ELBA	0.013/–	*^a^/n.s	1.010/–	*/n.s
	TDr	0.023/0.021	**^a^/**^a^		n.s./n.s
	WMR	0.028/0.028	*^a^/*^a^	1.130 / 1.901	*/*
	SI	0.023/0.020	**^a^/**^a^	0.885 / 0.574	*/*
Occipital right/left	SUVr		n.s./n.s		n.s./n.s
	ELBA		n.s./n.s		n.s./n.s
	TDr	0.019/0.019	**^a^/*^a^		n.s./n.s
	WMR	–/0.018	n.s./*^a^		n.s./n.s
	SI	0.021/0.026	**^a^/**^a^		n.s./n.s

^**^p < 0.001, *p < 0.05, n.s. *p* > 0.05

^a^Still significant (p < 0.05) after p-value correction for multiple comparisons with the Benjamini–Hochberg procedure for multiple testing

**Fig. 5:**
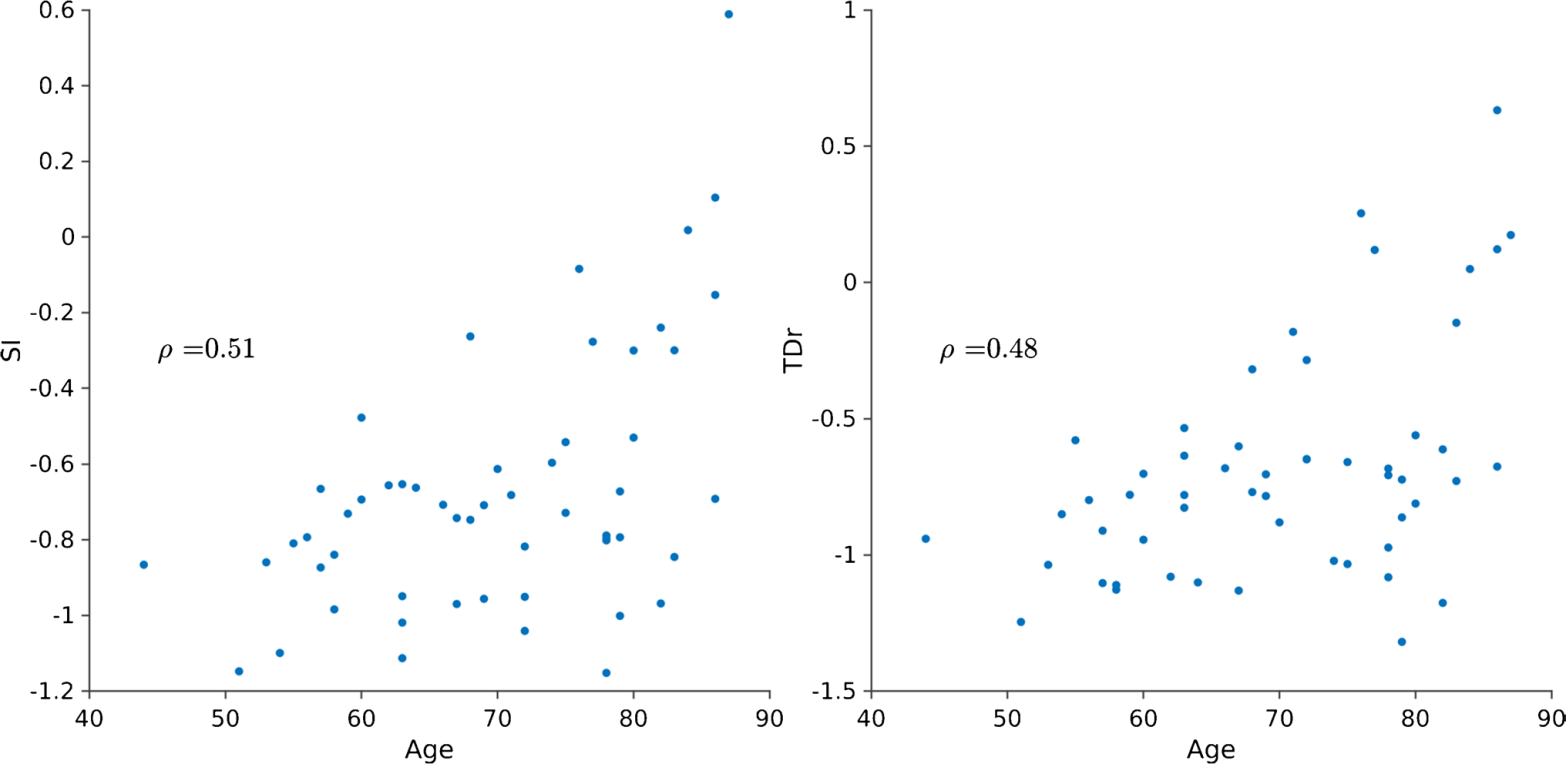
Scatter plot of age vs. whole-brain SI (left) and TDr (right) in the amyloid-negative subset. Both SI and TDr correlated significantly with age (*ρ* = 0.51 and *ρ* = 0.48, respectively)

At the global level, TDr, WMR, and SI were significantly associated with age (adjusted *p* < 0.05). Similarly, in each brain lobe (both right and left hemispheres) significant associations were observed between TDr, WMR and SI and age (adjusted *p* < 0.05). The only exception was WMR in the right occipital lobe, which was not related to age (*p* > 0.05). Linear relationships between SUVr, ELBA, and age were observed only in the right temporal lobes (adjusted *p* < 0.05). No associations between cortical thickness and the quantifiers survived at both the global and lobar levels. The regression slopes of each significant association were positive. Figure 5 shows the positive associations between the quantifiers including early acquisitions and age at the whole brain level.

## Discussion

The overall purpose of this study was to compare approaches for semi-quantitative analysis of amy-PET image data using different sources of information and with increasing levels of refinement.

Among the methods considered in this comparison, SI is the most comprehensive and the most complex as it takes into account the widest range of potentially confounding factors.

Like WMR, the SI requires dual time-point PET/MRI acquisition (or a PET/CT and a 3DT1 MPRAGE isotropic acquisition) and the construction of a patient-specific atlas [23].

Under particular conditions however (e.g., in case of low perfusion of the target region), the WMR denominator can become small, making this index numerically unstable. Moreover, inaccuracies in the segmentation of the WM in PET may increase its variability.

These considerations, despite the two methods using the same imaging data, led us to keep SI as the reference measure of Aβ against which the performances of SUVr [19], TDr [22], ELBA [20], and WMR [23] were evaluated.

At the whole-brain level, each quantifier showed excellent agreement with the visual assessment, so in terms of the binary classification there was substantial equivalence between the methods. Visual assessment showed that our dataset mainly comprised two distinct clusters: amyloid-negative (SI; *μ* = 0.01 ± 0.12) and amyloid-positive patients (SI; *μ* = 0.65 ± 0.19). A much larger dataset including subjects with prodromal AD stages could better elucidate the “gray zone” between positivity and negativity, and possibly heighten the differences among the methods.

The correlation analysis showed that there were strong associations between SI and every quantifier considered in this study, at both the regional and global levels. At the global level, WMR was the approach that most diverged from SI (confirmed by the dispersion analysis) as it considers the kinetics in a given cortical region compared with the kinetics in the WM of the same subject. Other quantifiers, however, use WM information, although only partially: ELBA measures the contrast between WM and GM, and TDr uses WM to normalize the counts on the hot spot. Only SUVr (at least the cortico-cerebellar implementation) focuses mainly on pure cortical distribution, without considering WM distribution. The fact that WMR correlates with the visual binary classification and with age in cases classified as qualitatively negative shows it to be a good metric, albeit based on different assumptions.

At the regional level, the differences between the quantifiers seem to be related to specific characteristics of the approaches. For example, a lack of agreement between ELBA and SI was found in the basal ganglia (central ROI). This may be explained by several factors: first, image registration does not rely on the accompanying MRI, and second, the WM/GM contrast — the ELBA’s backbone — is harder to identify in deep structures. SI, on the other hand, constructs a patient-specific atlas (based on the patient’s MR), which allows for a much more precise alignment of the basal ganglia, deep nuclei, and insula. These results might have relevance for imaging Down syndrome or genetic AD patients, as in these entities amyloid pathology is also present in basal ganglia areas.

As seen in the results section, SUVr, ELBA, TDr, and WMR all come close to SI despite differing in their nature and characteristics. By linearly combining these techniques we obtained scores (i.e., AVG1, AVG2, AVG3) closer to SI. Moreover, by repeating the analysis with AVG1, AVG2, and AVG3 calculated as unweighted averages, these combinations were found to be even closer to SI than the single approaches (see Supplementary Table 4). This suggests that the weights, calculated with respect to SI, only introduce an improvement factor and confirms the suitability of SI as the reference. If this were not the case, then combining different independent methods blind to the reference would not achieve greater closeness, and might even move away from it.

Even if scans come from a single center, they may exhibit heterogeneity that can differently impact the quantifiers (see Supplementary Fig. 2). The method-specific fluctuations observed on the distance from the SI suggest that a multi-method approach (i.e., the integration of different sources of information and/or independent techniques) is ideal. Indeed, we found that a combination of independent quantifiers provided better results than the individual quantifiers both in terms of correlation and distance from the chosen reference method. The analysis of the independence of SUVr, ELBA, and TDr confirms the observations of our group [22] using a different radiotracer.

Furthermore, it should be noted that, regardless of the analysis aim, the integration of structural imaging into the image registration and ROIs definition improves robustness, but with an additional complexity in processing.

In patients qualitatively classified as amyloid negative, the methods that included a correction for blood flow (SI, WMR, and TDr) were able to identify the physiologic accumulation of amyloid with age, showing that a metric that includes the early phase is more accurate (i.e., that includes information on blood flow and hence on neurodegeneration).

### Further considerations on the quantifier choice

**Fig. 6:**
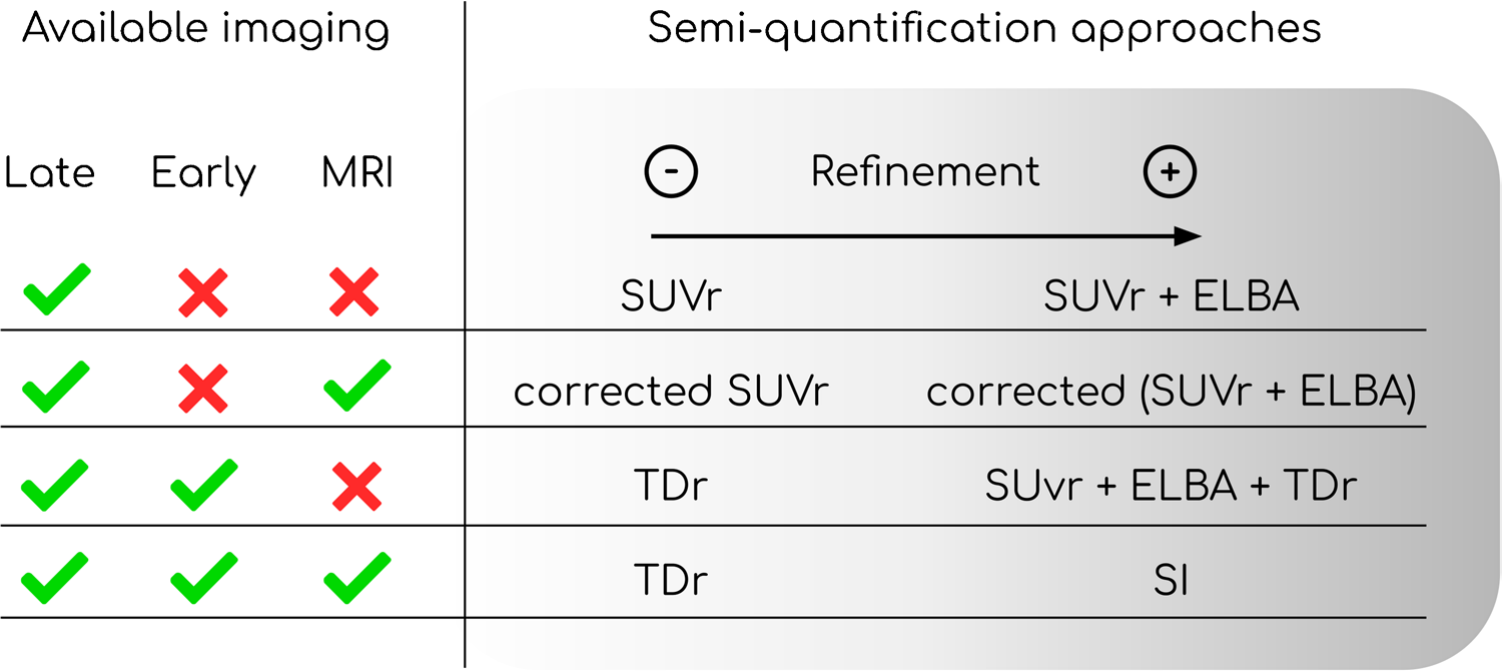
Possible choices of amy-PET semi-quantification approach based on imaging data availability and analysis refinement. T1, MRI 3DT1; Late, late static amy-PET acquisition; Early, early static amy-PET acquisition; Corrected, atrophy-corrected analysis

A first discriminating factor in choosing a suitable semi-quantification method is the imaging data availability (i.e., early, late acquisition and MRI). Depending on it (and secondarily, on the desired level of analysis refinement), different strategies can be chosen. The results of our study suggest, for example, that a raw binary evaluation of late scan can already be achieved with the SUVr alone. Further improvement of late scan analysis could be provided by integrating ELBA. A graphical representation of the possible approaches considering different levels of refinement is provided in Fig. 6. From this perspective, the addition of an early scan would allow the use of TDr. Instead, if higher complexity is possible (MRI also available) then the SI is the optimal choice.

Another relevant aspect for the quantifier choice is the clinical question. Our results suggest that, if the aim is a binary classification, then the methods using the late scan alone are already sufficiently informative. Therefore, for this purpose, the SUVr — a widely used, well-documented approach — appears to be a suitable solution. Moreover, both this study and that of Müller and colleagues [30] show that the SUVr performs equally well in discriminating visually positive and negative scans, regardless of implementation and normalization procedure (see the Supplementary Table 5).

In contrast, the ability of quantifiers integrating early acquisition to detect subtle information (such as amyloid load due to aging) makes them more suitable for more refined analyses. The early acquisition not only allows for blood flow correction, but also provides independent information with added clinical value, irrespective of the Aβ status. Indeed, many studies suggest that the early acquisition provides a valid surrogate marker for neuronal injury which could avoid the need for additional ^18^F-FDG PET assessment in diagnosis of dementia [31–34].

Among the quantifiers that derive information from dual time-window protocols, the TDr seems to represent an acceptable compromise between complexity and accuracy of the analysis.

## Conclusions

The quantifiers we evaluated (SUVr, ELBA, TDr, and WMR), came close to SI, chosen as the reference method, even though they are different in nature and in their characteristics. If we were to single out one method, it would be TDr (accepting some imprecision in the deep structures), which appears to be accurate (deriving information from dual time points) and easier to implement than SI (no CT or MR needed). However, this study suggests that a combination of independent quantifiers yields better results than the individual approaches, both in terms of correlation and distance from the chosen reference method. Efforts should therefore be made towards developing multi-classifier systems to measure amyloid pathology in vivo by PET. Moreover, the ability of some quantifiers (TDr, WMR, and SI) to depict variations in brain amyloid load with age in subjects assessed qualitatively as amyloid-negative demonstrates the goodness of the estimate and their potential for identifying subtle variations in amyloid load compared with standard methods (such as SUVr).

## Supplementary Information

Below is the link to the electronic supplementary material.

Supplementary file1 (DOCX 3.34 KB)

Supplementary file2 (PNG 1408 KB)

Supplementary file3 (PNG 5498 KB)

## References

[1] Jack CR, Bennett DA, Blennow K, Carrillo MC, Dunn B, Haeberlein SB, Holtzman DM, Jagust W, Jessen F, Karlawish J, Liu E, Molinuevo JL, Montine T, Phelps C, Rankin KP, Rowe CC, Scheltens P, Siemers E, Snyder HM, Sperling R, Elliott C, Masliah E, Ryan L, Silverberg N (2018). NIA-AA Research Framework: toward a biological definition of Alzheimer’s disease. Alzheimer’s Dement.

[2] Barthel H, Sabri O (2017). Clinical Use and utility of amyloid imaging. J Nucl Medicine.

[3] Fantoni ER, Chalkidou A, O’Brien JT, Farrar G, Hammers A (2018). A Systematic review and aggregated analysis on the impact of amyloid PET Brain imaging on the diagnosis diagnostic confidence and management of patients being evaluated for Alzheimer’s disease. J Alzheimers Dis.

[4] Jansen WJ, Ossenkoppele R, Knol DL, Tijms BM, Scheltens P, Verhey FRJ, Visser PJ, Aalten P, Aarsland D, Alcolea D, Alexander M, Almdahl IS, Arnold SE, Baldeiras I, Barthel H, van Berckel BNM, Bibeau K, Blennow K, Brooks DJ, van Buchem MA, Camus V, Cavedo E, Chen K, Chetelat G, Cohen AD, Drzezga A, Engelborghs S, Fagan AM, Fladby T, Fleisher AS, van der Flier WM, Ford L, Förster S, Fortea J, Foskett N, Frederiksen KS, Freund-Levi Y, Frisoni GB, Froelich L, Gabryelewicz T, Gill KD, Gkatzima O, Gómez-Tortosa E, Gordon MF, Grimmer T, Hampel H, Hausner L, Hellwig S, Herukka S-K, Hildebrandt H, Ishihara L, Ivanoiu A, Jagust WJ, Johannsen P, Kandimalla R, Kapaki E, Klimkowicz-Mrowiec A, Klunk WE, Köhler S, Koglin N, Kornhuber J, Kramberger MG, Van Laere K, Landau SM, Lee DY, de Leon M, Lisetti V, Lleó A, Madsen K, Maier W, Marcusson J, Mattsson N, de Mendonça A, Meulenbroek O, Meyer PT, Mintun MA, Mok V, Molinuevo JL, Møllergård HM, Morris JC, Mroczko B, Van der Mussele S, Na DL, Newberg A, Nordberg A, Nordlund A, Novak GP, Paraskevas GP, Parnetti L, Perera G, Peters O, Popp J, Prabhakar S, Rabinovici GD, Ramakers IHGB, Rami L, Resende de Oliveira C, Rinne JO, Rodrigue KM, Rodríguez-Rodríguez E, Roe CM, Rot U, Rowe CC, Rüther E, Sabri O, Sanchez-Juan P, Santana I, Sarazin M, Schröder J, Schütte C, Seo SW, Soetewey F, Soininen H, Spiru L, Struyfs H, Teunissen CE, Tsolaki M, Vandenberghe R, Verbeek MM, Villemagne VL, Vos SJB, van Waalwijk LJC, van Doorn G, Waldemar A, Wallin ÅK, Wallin J, Wiltfang DA, Wolk M. Zboch, Zetterberg H (2015). Prevalence of cerebral amyloid pathology in persons without dementia. JAMA.

[5] Jack CR, Wiste HJ, Weigand SD, Rocca WA, Knopman DS, Mielke MM, Lowe VJ, Senjem ML, Gunter JL, Preboske GM, Pankratz VS, Vemuri P, Petersen RC (2014). Age-specific population frequencies of cerebral β-amyloidosis and neurodegeneration among people with normal cognitive function aged 50–89 years: a cross-sectional study. Lancet Neurol.

[6] Rodrigue KM, Kennedy KM, Devous MD, Rieck JR, Hebrank AC, Diaz-Arrastia R, Mathews D, Park DC (2012). β-Amyloid burden in healthy aging: regional distribution and cognitive consequences. Neurology.

[7] Gonneaud J, Arenaza-Urquijo EM, Mézenge F, Landeau B, Gaubert M, Bejanin A, de Flores R, Wirth M, Tomadesso C, Poisnel G, Abbas A, Desgranges B, Chételat G (2017). Increased florbetapir binding in the temporal neocortex from age 20 to 60 years. Neurology.

[8] Collij LE, Konijnenberg E, Reimand J, Kate MT, Braber AD, Alves IL, Zwan M, Yaqub M, van Assema DM, Wink AM, Lammertsma AA, Scheltens P, Visser PJ, Barkhof F, van Berckel BN (2019). Assessing amyloid pathology in cognitively normal subjects using 18 F-Flutemetamol PET: comparing visual reads and quantitative methods. J Nucl Med.

[9] Nayate AP, Dubroff JG, Schmitt JE, Nasrallah I, Kishore R, Mankoff D, Pryma DA (2015). Use of standardized uptake value ratios decreases interreader variability of [18F] florbetapir PET brain scan interpretation. Am J Neuroradiol.

[10] A Chincarini, E Peira, S Morbelli, M Pardini, M Bauckneht, J Arbizu, M Castelo-Branco, K Büsing, A de Mendonça, M Didic, M Dottorini, S Engelborghs, C Ferrarese, G Frisoni, V Garibotto, E Guedj, L Hausner, J Hugon, J Verhaeghe, P Mecocci, M Musarra, M Queneau, M Riverol, I Santana, U Guerra and F Nobili. Semi-quantification and grading of amyloid PET: a project of the european Alzheimer’s Disease Consortium (EADC). NeuroImage Clin. 2019; 23:101846. 10.1016/j.nicl.2019.101846. 10.1016/j.nicl.2019.101846 PMC6514268 31077984

[11] Sevigny J, Chiao P, Bussière T, Weinreb PH, Williams L, Maier M, Dunstan R, Salloway S, Chen T, Ling Y, O’Gorman J, Qian F, Arastu M, Li M, Chollate S, Brennan MS, Quintero-Monzon O, Scannevin RH, Arnold HM, Engber T, Rhodes K, Ferrero J, Hang Y, Mikulskis A, Grimm J, Hock C, Nitsch RM, Sandrock A (2016). The antibody aducanumab reduces Aβ plaques in Alzheimer’s disease. Nature.

[12] Egan MF, Kost J, Voss T, Mukai Y, Aisen PS, Cummings JL, Tariot PN, Vellas B, van Dyck CH, Boada M, Zhang Y, Li W, Furtek C, Mahoney E, Harper Mozley L, Mo Y, Sur C, Michelson D (2019). Randomized Trial of Verubecestat for prodromal Alzheimer’s DISEASe. N Engl J Med.

[13] Salloway S, Sperling R, Fox NC, Blennow K, Klunk W, Raskind M, Sabbagh M, Honig LS, Porsteinsson AP, Ferris S, Reichert M, Ketter N, Nejadnik B, Guenzler V, Miloslavsky M, Wang D, Lu Y, Lull J, Tudor IC, Liu E, Grundman M, Yuen E, Black R, Brashear HR (2014). Two phase 3 trials of bapineuzumab in mild-to-moderate Alzheimer’s disease. N Engl J Med.

[14] Honig LS, Vellas B, Woodward M, Boada M, Bullock R, Borrie M, Hager K, Andreasen N, Scarpini E, Liu-Seifert H, Case M, Dean RA, Hake A, Sundell K, Poole Hoffmann V, Carlson C, Khanna R, Mintun M, DeMattos R, Selzler KJ, Siemers E (2018). Trial of solanezumab for mild dementia due to Alzheimer’s disease. N Engl J Med.

[15] Vandenberghe R, Rinne JO, Boada M, Katayama S, Scheltens P, Vellas B, Tuchman M, Gass A, Fiebach JB, Hill D, Lobello K, Li D, McRae T, Lucas P, Evans I, Booth K, Luscan G, Wyman BT, Hua L, Yang L, Brashear HR, Black RS (2016). Bapineuzumab for mild to moderate Alzheimer’s disease in two global, randomized, phase 3 trials. Alzheimers Res Ther.

[16] Grothe MJ, Barthel H, Sepulcre J, Dyrba M, Sabri O, Teipel SJ (2017). In vivo staging of regional amyloid deposition. Neurology.

[17] Mattsson N, Palmqvist S, Stomrud E, Vogel J, Hansson O (2019). Staging β -Amyloid Pathology with amyloid positron emission tomography. JAMA Neurol.

[18] Sakr FA, Grothe MJ, Cavedo E, Jelistratova I, Habert M-O, Dyrba M, Gonzalez-Escamilla G, Bertin H, Locatelli M, Lehericy S, Teipel S, Dubois B, Hampel H (2019). Applicability of in vivo staging of regional amyloid burden in a cognitively normal cohort with subjective memory complaints: the INSIGHT-preAD study. Alzheimers Res Ther.

[19] Kinahan PE, Fletcher JW (2010). "Positron emission tomography-computed tomography standardized uptake values in clinical practice and assessing response to Therapy" Seminars in Ultrasound. CT and MRI.

[20] Chincarini A, Sensi F, Rei L, Bossert I, Morbelli S, Guerra UP, Frisoni G, Padovani A, Nobili F (2016). Standardized uptake value ratio-independent evaluation of brain amyloidosis. J Alzheimers Dis.

[21] Whittington A, Gunn RN (2019). Amyloid Load: a more sensitive biomarker for amyloid imaging. J Nucl Med.

[22] Chincarini A, Peira E, Corosu M, Morbelli S, Bauckneht M, Capitanio S, Pardini M, Arnaldi D, Vellani C, D’Ambrosio D, Garibotto V, Assal F, Paghera B, Savelli G, Stefanelli A, Guerra UP, Nobili F (2020). A kinetics-based approach to amyloid PET semi-quantification. Eur J Nucl Med Mol Imaging.

[23] Cecchin D, Barthel H, Poggiali D, Cagnin A, Tiepolt S, Zucchetta P, Turco P, Gallo P, Frigo AC, Sabri O, Bui F (2017). A new integrated dual time-point amyloid PET/MRI data analysis method. Eur J Nucl Med Mol Imaging.

[24] Schmidt ME, Chiao P, Klein G, Matthews D, Thurfjell L, Cole PE, Margolin R, Landau S, Foster NL, Mason NS, De Santi S, Suhy J, Koeppe RA, Jagust W (2015). The influence of biological and technical factors on quantitative analysis of amyloid PET: points to consider and recommendations for controlling variability in longitudinal data. Alzheimers Dement.

[25] Bullich S, Barthel H, Koglin N, Becker GA, De Santi S, Jovalekic A, Stephens AW, Sabri O (2018). Validation of noninvasive tracer kinetic analysis of 18 F-Florbetaben PET using a dual–time-window acquisition protocol. J Nucl Med.

[26] Garibotto V, Albert NL, Barthel H, van Berckel B, Boellaard R, Brendel M, Cecchin D, Ekmekcioglu O, van de Giessen E, Guedj E, Lammerstma AA, Semah F, Traub-Weidinger T, Van Weehaeghe D, Morbelli S (2021). The approval of a disease-modifying treatment for Alzheimer’s disease: impact and consequences for the nuclear medicine community. Eur J Nucl Med Mol Imaging.

[27] European Medicines Agency. Neuraceq: florbetaben (18F). Product information. 1999. https://www.ema.europa.eu/en/medicines/human/EPAR/neuraceq. Accessed 10 Jan 2022.

[28] Minoshima S, Drzezga AE, Barthel H, Bohnen N, Djekidel M, Lewis DH, Mathis CA, McConathy J, Nordberg A, Sabri O, Seibyl JP, Stokes MK, Van Laere K (2016). SNMMI Procedure standard/EANM practice guideline for amyloid PET imaging of the brain 1.0. J Nucl Med.

[29] Fischl B (2012). FreeSurfer. NeuroImage.

[30] Müller EG, Stokke C, Stokmo HL, Edwin TH, Knapskog AB, Revheim ME (2022). Evaluation of semi-quantitative measures of 18 F-flutemetamol PET for the clinical diagnosis of Alzheimer’s disease. Quant Imaging Med Surg.

[31] Chen YJ, Rosario BL, Mowrey W, Laymon CM, Lu X, Lopez OL, Klunk WE, Lopresti BJ, Mathis CA, Price JC (2015). Relative 11C-pib delivery as a proxy of relative CBF: quantitative evaluation using single-session 15O-water and 11C-pib PET. J Nucl Med.

[32] Tiepolt S, Hesse S, Patt M, Luthardt J, Schroeter ML, Hoffmann KT, Weise D, Gertz HJ, Sabri O, Barthel H (2016). Early [(18)F]florbetaben and [(11)C]PiB PET images are a surrogate biomarker of neuronal injury in Alzheimer’s disease. Eur J Nucl Med Mol Imaging.

[33] Daerra S, Brendel M, Zach C, Mille E, Schilling D, Zacherl MJ, Bürger K, Danek A, Pogarell O, Schildan A, Patt M, Barthel H, Sabri O, Bartenstein P, Rominger A (2017). Evaluation of early-phase [18F]-florbetaben PET acquisition in clinical routine cases. Neuroimage: Clin.

[34] Schmitt J, Palleis C, Sauerbeck J, Unterrainer M, Harris S, Prix C, Weidinger E, Katzdobler S, Wagemann O, Danek A, Beyer L, Rauchmann BS, Rominger A, Simons M, Bartenstein P, Perneczky R, Haass C, Levin J, Höglinger GU, Brendel M, the German Imaging Initiative for Tauopathies (GII4T) (2021). Dual-phase β-amyloid PET Captures neuronal injury and amyloidosis in corticobasal syndrome. Front Aging Neurosci.

