# Supplementary material for: A comparison of advanced semi-quantitative amyloid PET analysis methods
Source: Eur J Nucl Med Mol Imaging. 2022 Jun 2;49(12):4097–108. doi: 10.1007/s00259-022-05846-1 (PMC9525368; doi:10.1007/s00259-022-05846-1)
Supplement: Supplementary file 1 — Supplementary file1 (DOCX 3.34 KB) [file 259_2022_5846_MOESM1_ESM.docx]

Supplementary materials

A comparison of advanced semi-quantitative amyloid PET analysis methods.

submitted to “European Journal of Nuclear Medicine and Molecular Imaging”

E. Peira^1,2^*, D. Poggiali^3^, M. Pardini^2,4^, H. Barthel^5^, O. Sabri^5^, S. Morbelli^4,6^, A. Cagnin^7^, A. Chincarini^1^, D. Cecchin^3,8^.

**[Affiliations]**

[1] INFN – National Institute of Nuclear Physics, via Dodecaneso 33, I-16146 Genoa, Italy

[2] Department of Neuroscience, Rehabilitation, Ophthalmology, Genetics, Child and Maternal Health (DINOGMI), University of Genoa, Genoa, Italy

[3] PNC - Padua Neuroscience Center, University of Padua, Padua, Italy

[4] IRCCS Ospedale Policlinico San Martino, Genoa, Italy

[5] Department of Nuclear Medicine, University Hospital Leipzig,

Leipzig, Germany

[6] Nuclear Medicine Unit, Department of Health Sciences (DISSAL), University of Genoa, Genoa, Italy

[7] Neurology Unit, Department of Neurology, University Hospital of Padua, Italy

[8] Nuclear Medicine Unit, Department of Medicine - DIMED, University Hospital of Padua, Italy

*Corresponding author

email: enrico.peira@gmail.com

# Supplementary Tables

Supplementary Table 1: Diagnoses of the patients grouped by visual classification. MCI: mild cognitive impairment; LBD: Lewy body dementia; bvFTD: behavioral variant frontotemporal degeneration; probADD: probable AD dementia; possADD: possible AD dementia; atypAD: atypical AD; mixDem: mixed-type dementia.

| diagnosis | Visual classification | |
| --- | --- | --- |
|  | negative | positive |
| MCI | 23 | 10 |
| LBD | 1 | - |
| bvFTD | 4 | 1 |
| probADD | 8 | 9 |
| possADD | 14 | 7 |
| atypAD | 2 | 3 |
| mixDem | 2 | 1 |

Supplementary Table 2: Dispersions of the quantifiers (and of their linear combinations) from SI at the brain and lobar levels: results of the Bland-Altman analysis. The bootstrapped divergences from SI are expressed as average σ and the 95% confidence interval.

|  | **SUVr** | **ELBA** | **TDr** | **WMR** | **AVG1** | **AVG2** | **AVG3** |
| --- | --- | --- | --- | --- | --- | --- | --- |
| Whole brain | .31  [.3 - .31] | .39  [.39 - .4] | .32  [.32 - .33] | .57  [.56 - .58] | .25  [.25 - .25] | .24  [.24 - .25] | .25  [.25 - .26] |
| Frontal right | .32  [.32 - 32] | .42  [.42 - 43] | .31  [.31 - .31] | .33  [.33 - .33] | .29  [.29 - .3] | .25  [.25 - .26] | .24  [.23 - .24] |
| Frontal  left | .32  [.32 - .32] | .41  [.41 - .41] | .33  [.33 - .34] | .33  [.33 - .33] | .29  [.29 - .29] | .27  [.27 - .27] | .25  [.25 - .25] |
| Parietal  right | .34  [.34 - .35] | .39  [.39 - .4] | .32  [.32 - .32] | .31  [.30 - .31] | .29  [.29 - .29] | .27  [.26 - .27] | .23  [.23 - .24] |
| Parietal  left | .36  [.36 - .36] | .4  [.4 - .4] | .35  [.35 - .35] | .31  [.31 - .31] | .31  [.31 - .31] | .29  [.29 - .29] | .25  [.25 - .25] |
| Temporal  right | .32  [.32 - .32] | .43  [.42 - .43] | .39  [.39 - .39] | .54  [.54 - .55] | .29  [.28 - .29] | .27  [.27 - .27] | .25  [.25 - .25] |
| Temporal left | .36  [.36 - .36] | .46  [.45 - .46] | .41  [.41 - .41] | .76  [.75 - .78] | .32  [.32 - .33] | .31  [.3 - .31] | .31  [.31 - .32] |
| Occipital  right | .46  [.46 - .47] | .46  [.45 - .46] | .36  [.36 - .37] | .44  [.44 - .45] | .37  [.37 - .37] | .34  [.34 - .34] | .32  [.32 - .33] |
| Occipital left | .51  [.51 - .51] | .48  [.48 - .49] | .44  [.44 - .44] | .44  [.44 - .44] | .43  [.42 - .43] | .40  [.40 - .40] | .36  [.35 - .36] |
| Central right | .64  [.63 - .64] | 1.06  [1.05 - 1.07] | .71  [.71 - .72] | .78  [.77 - .79] | .65  [.64 - .65] | .57  [.57 - .58] | .54  [.54 - .55] |
| Central  left | .61  [.61 - .62] | 1.03  [1.02 - 1.04] | .68  [.67 - .68] | .74  [.74 - .75] | .63  [.63 - .64] | .58  [.57 - .58] | .53  [.53 - .54] |

Supplementary Table 3: Regional and global bootstrapped performances (expressed as average AUC and CI 95%) of the quantifiers and their linear combinations vs. visual assessment.

|  | **SUVr** | **ELBA** | **TDr** | **WMR** | **AVG1** | **AVG2** | **AVG3** | **SI** |
| --- | --- | --- | --- | --- | --- | --- | --- | --- |
| Whole brain | .99  [.9 - 1] | 1  [1 - 1] | 1  [.98 - 1] | .98  [.93 - .99] | 1  [.98 - 1] | 1  [.99 - 1] | 1  [.99 - 1] | 1  [.98 - 1] |
| Frontal right | .98  [.91 - 1] | .99  [.97 - 1] | .99  [.97 - 1] | .99  [.96 - 1] | .99  [.95 - 1] | .99  [.97 - 1] | .99  [.97 - 1] | .99  [.96 - 1] |
| Frontal  left | .99  [.93 - 1] | 1  [1 - 1] | 1  [.99 - 1] | 1  [.97 - 1] | 1  [.96 - 1] | 1  [.99 - 1] | 1  [.99 - 1] | 1  [.97 - 1] |
| Parietal  right | .99  [.94 - 1] | 1  [.99 - 1] | .99  [.97 - 1] | .99  [.95 - 1] | 1  [.98 - 1] | 1  [.97 - 1] | .99  [.97 - 1] | .99  [.97 - 1] |
| Parietal  left | .99  [.96 - 1] | 1  [1 - 1] | .99  [.97 - 1] | .99  [.96 - 1] | 1  [.99 - 1] | 1  [.99 - 1] | 1  [.99 - 1] | 1  [.96 - 1] |
| Temporal  right | .97  [.87 - 1] | .99  [.97 - 1] | .99  [.96 - 1] | .95  [.83 - .99] | .99  [.95 - 1] | .99  [.97 - 1] | .99  [.97 - 1] | .99  [.96 - 1] |
| Temporal left | .99  [.95 - 1] | 1  [1 - 1] | .99  [.98 - 1] | .96  [.89 - .99] | 1  [.99 - 1] | 1  [1 - 1] | 1  [1 - 1] | .99  [.97 - 1] |
| Occipital  right | .98  [.94 - 1] | .98  [.93 - 1] | .99  [.96 - 1] | .93  [.84 - .98] | .99  [.96 - 1] | .99  [.97 - 1] | .99  [.96 - 1] | .97  [.92 - 99] |
| Occipital left | .98  [.92 - 1] | .96  [.88 - .99] | .97  [.91 - .99] | .91  [.81 - .96] | .98  [.94 - 1] | .98  [.95 - 1] | .97  [.93 - .99] | .93  [.85 - .98] |
| Central right | .96  [.83 - .99] | .71  [.58 - .82] | .94  [.88 - .97] | .9  [.77 - .96] | .95  [.87 - .98] | .97  [.91 - .99] | .97  [.91 - .99] | .97  [.91 - .99] |
| Central  left | .96  [.85 - .99] | .79  [.68 - .87] | .98  [.95 - 1] | .94  [.86 - .98] | .95  [.85 - .99] | .98  [.93 - 1] | .99  [.97 - 1] | .96  [.89 - .98] |

Supplementary Table 4: Bootstrapped dispersions, Pearson correlation coefficients, and bootstrapped performances of the three combinations AVG1, AVG2 and AVG3 calculated as the unweighted, arithmetic means of the quantifiers.

|  | Dispersion (σ) | | | Correlation (ρ) | | | AUC | | |
| --- | --- | --- | --- | --- | --- | --- | --- | --- | --- |
|  | **AVG1** | **AVG2** | **AVG3** | **AVG1** | **AVG2** | **AVG3** | **AVG1** | **AVG2** | **AVG3** |
| Whole brain | .26  [.26 - .26] | .25  [.25 - .25] | .28  [.27 - .28] | .96 | .97 | .96 | 1  [.97 - 1] | 1  [.98 - 1] | 1  [.99 - 1] |
| Frontal right | .30  [.30 - .30] | .26  [.26 - .26] | .24  [.24 - .24] | .95 | .96 | .97 | .99  [.93 - 1] | .99  [.97 - 1] | .99  [.97 - 1] |
| Frontal  left | .29  [.29 - .30] | .28  [.27 - .28] | .26  [.26 - .26] | .95 | .96 | .97 | 1  [.97 - 1] | 1  [.99 - 1] | 1  [.99 - 1] |
| Parietal  right | .29  [.29 - .30] | .27  [.27 - .27] | .24  [.23 - .24] | .95 | .96 | .97 | .1  [.98 - 1] | .1  [.98 - 1] | .99  [.97 - 1] |
| Parietal  left | .31  [.31 - .31] | .29  [.29 - .29] | .25  [.25 - .25] | .95 | .96 | .97 | 1  [.99 - 1] | 1  [.99 - 1] | 1  [.99 - 1] |
| Temporal  right | .29  [.29 - .30] | .28  [.27 - .28] | .26  [.26 - .26] | .95 | .96 | .96 | .99  [.95 - 1] | .99  [.96 - 1] | .99  [.97 - 1] |
| Temporal left | .33  [.33 - .33] | .31  [.31 - .32] | .35  [.34 - .35] | .94 | .95 | .94 | 1  [.99 - 1] | 1  [1 - 1] | 1  [1 - 1] |
| Occipital  right | .37  [.37 - .37] | .34  [.34 - .35] | .33  [.33 - .33 | .93 | .94 | .94 | .99  [.98 - 1] | .99  [.96 - 1] | .99  [.96 - .99] |
| Occipital left | .42  [.42 - .42] | .40  [.40 - .40] | .36  [.35 - .36] | .90 | .92 | .93 | .98  [.94 - 1] | .98  [.94 - 1] | .98  [.92 - .99] |
| Central right | .70  [.70 - .71] | .60  [.60 - .71] | .56  [.56 - .57] | .72 | .79 | .82 | .92  [.83 - .97] | .95  [.87 - .99] | .96  [.90 - .99] |
| Central  left | .68  [.68 - .69] | .61  [.60 - .61] | .56  [.55 - .56] | .73 | .79 | .82 | .94  [.84 - .98] | .97  [.91 - .99] | .99  [.97 - 1] |

Supplementary Table 5: Agreement, expressed as AUC, with visual classification of different SUVr implementation (whole-brain analysis) reported by Müller and colleagues [30]. As one may notice, these scores are comparable with results of our study (obtained from both SUVr and other quantifiers). SUVr pons: SUVr normalized to pons; SUVr cer: SUVr normalized to cerebellar cortex.

| Semi-quantitative measure | Software | AUC |
| --- | --- | --- |
| SUVr pons | SyngoVia | 0.99 |
|  | CortexID | 0.99 |
|  | PMOD | 0.99 |
| SUVr cer | SyngoVia | 0.99 |
|  | CortexID | 0.99 |
|  | PMOD | 0.99 |

# Supplementary Figures


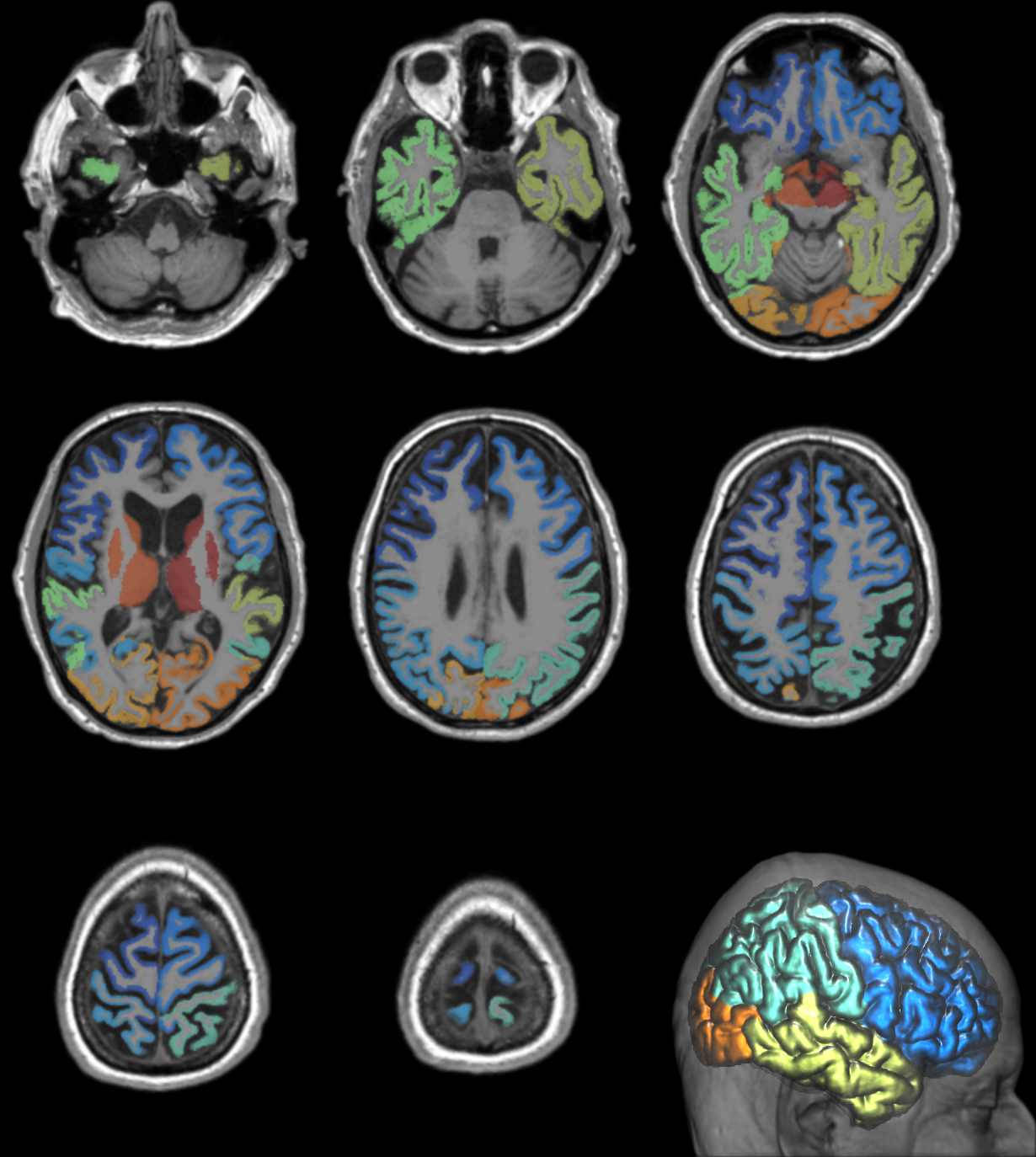


Supplementary Figure 1: Lobular parcellation of the brain adopted for this study. Each hemisphere (the right hemisphere is shown on the bottom right of the figure) was divided into 5 ROIs: frontal (blue), parietal (turquoise), temporal (yellow), occipital (orange), central structures (dark red).


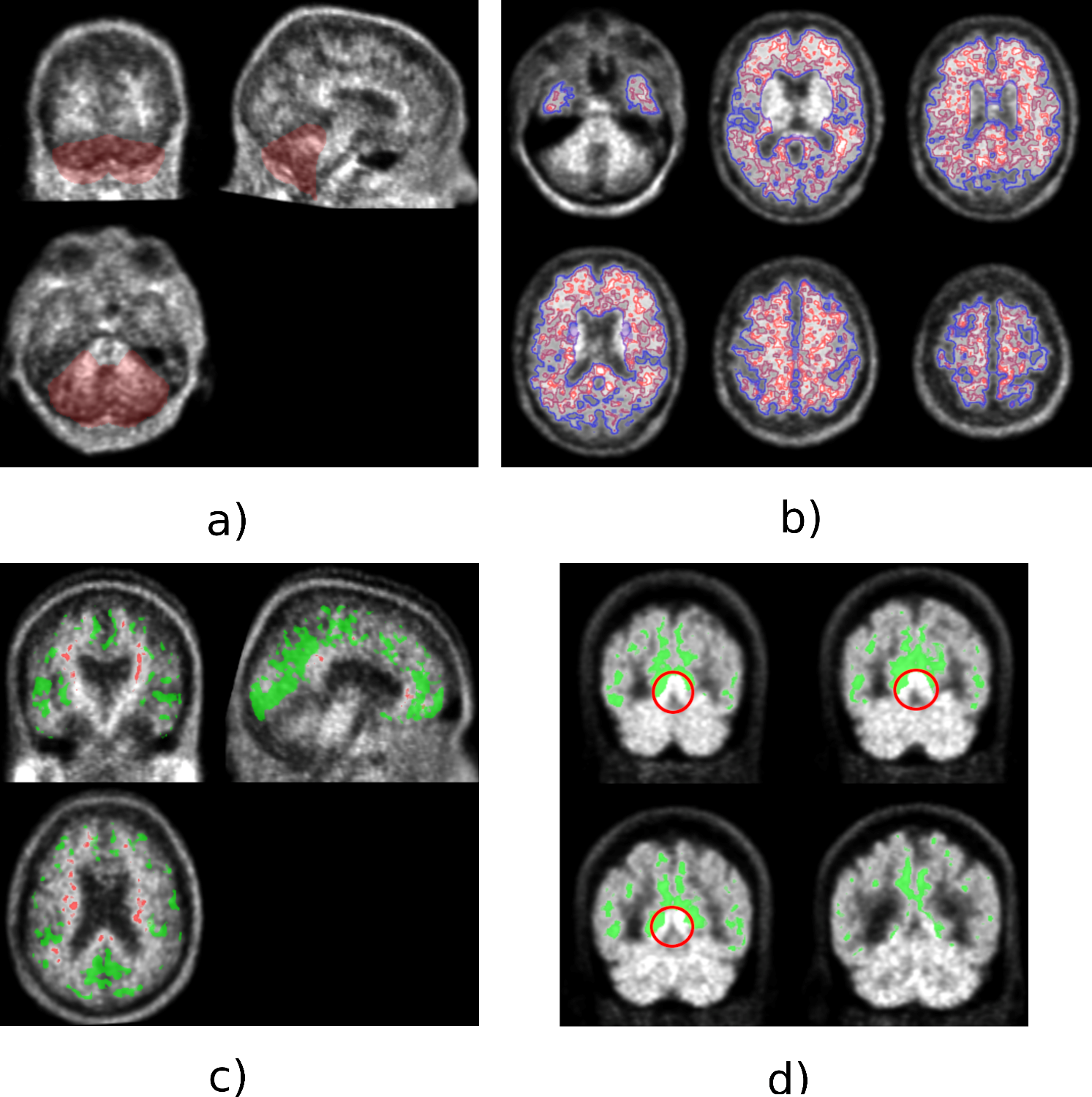
Supplementary Figure 2: Examples of cases semi-quantified with specific methods whose divergence from SI falls outside the 95% confidence interval of the Bland-Altmann analysis.

Coronal, sagittal, and axial view of a standard late acquisition analyzed with whole-brain SUVr (a). The red area delineates the cerebellar reference ROI. In this case, a misalignment of the cerebellar ROI can be observed. This inaccuracy, which is due to the use of pre-segmented ROIs, can be countered by integrating structural imaging into the pre-processing steps.

Iso-intensity partition illustration on an axial projection of a standard late acquisition analyzed with ELBA (b). The radiomic texture (geometric features) extracted by ELBA are sensitive to image quality that affect iso-intensity surface complexity, generating a more fluctuating score especially in positive scans.

c) and d) refers to a case analyzed with TDr. The late acquisition of this case is affected by movement artifact which may distort the counts distribution (c). This could affect, not only the co-registration of the early/late scan, but also the definition of the normalization ROI (red area). In this case we observe that the normalization region is composed of many small regions that are more prone to fluctuations and noise. Moreover, an incorrect realignment may have caused a partial exclusion of the area of highest perfusion (circled area) from the target region (green area) delineated on the early scan (d).
