# Supplementary figures and images for: A comparison of advanced semi-quantitative amyloid PET analysis methods
Source: Eur J Nucl Med Mol Imaging. 2022 Jun 2;49(12):4097–108. doi: 10.1007/s00259-022-05846-1 (PMC9525368; doi:10.1007/s00259-022-05846-1)

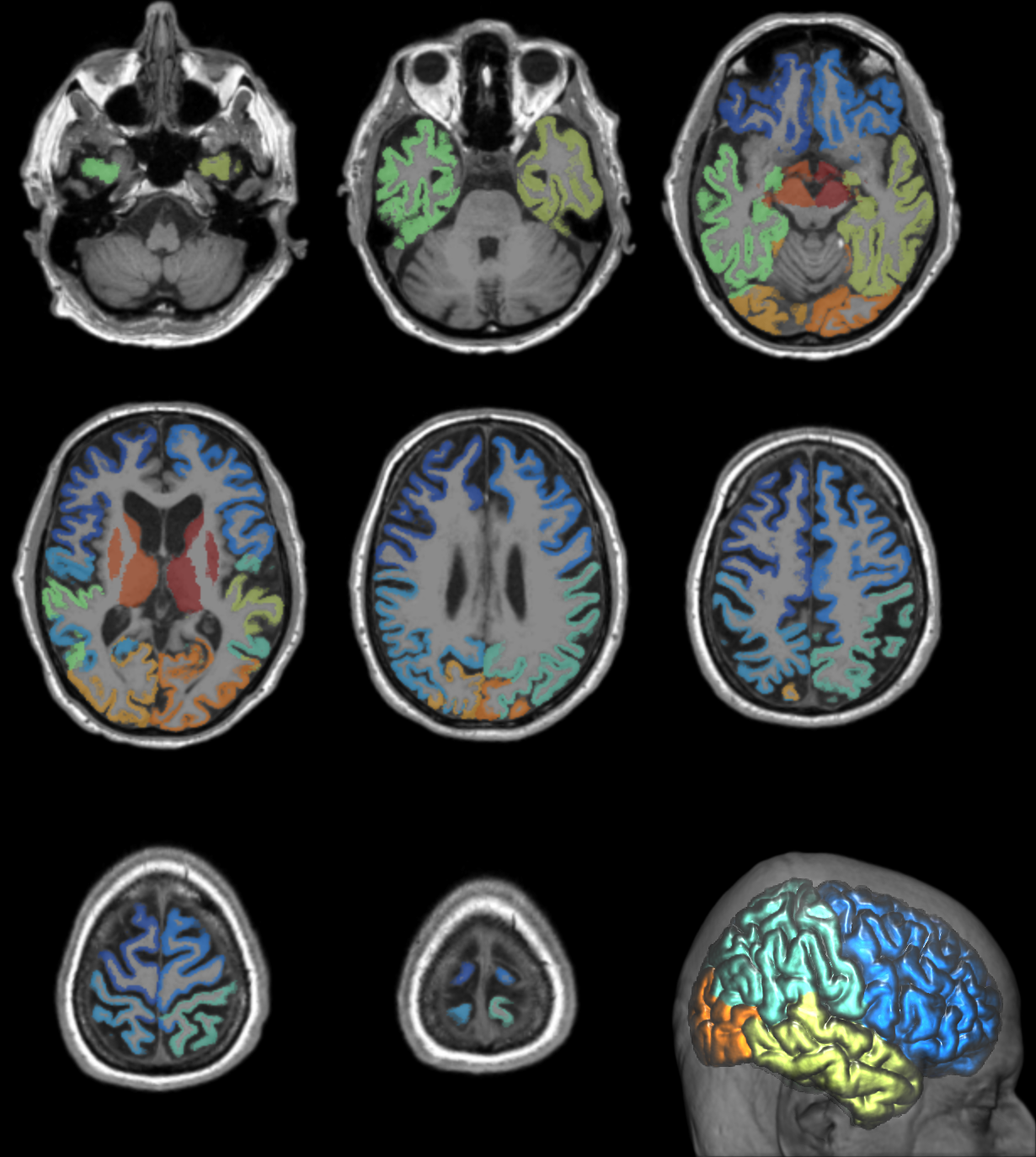

Supplement: Supplementary file 2 — Supplementary file2 (PNG 1408 KB) [file 259_2022_5846_MOESM2_ESM.png]

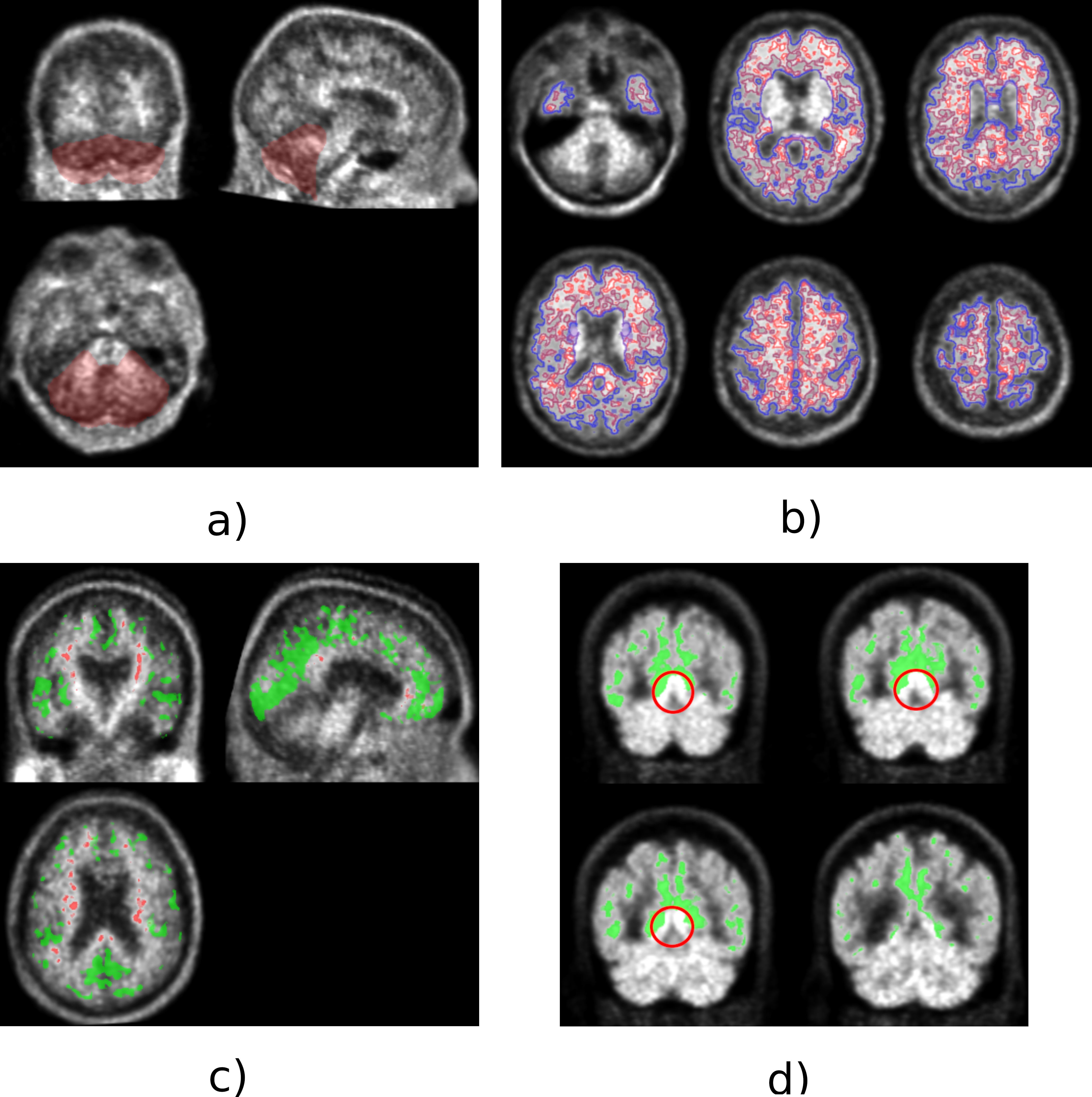

Supplement: Supplementary file 3 — Supplementary file3 (PNG 5498 KB) [file 259_2022_5846_MOESM3_ESM.png]
